# High‐resolution imaging of organic pharmaceutical crystals by transmission electron microscopy and scanning moiré fringes

**DOI:** 10.1111/jmi.12866

**Published:** 2020-02-18

**Authors:** M. S'ARI, N. KONIUCH, R. BRYDSON, N. HONDOW, A. BROWN

**Affiliations:** ^1^ School of Chemical and Process Engineering, University of Leeds Leeds UK

**Keywords:** Bright‐field STEM, High‐resolution TEM, Low dose, Organic crystals, Scanning moiré fringes

## Abstract

Formulation processing of organic crystalline compounds can have a significant effect on drug properties, such as dissolution rate or tablet strength/hardness. Transmission electron microscopy (TEM) has the potential to resolve the atomic lattice of these crystalline compounds and, for example, identify the defect density on a particular crystal face, provided that the sensitivity of these crystals to irradiation by high‐energy electrons can be overcome. Here, we acquire high‐resolution (HR) lattice images of the compound furosemide using two different methods: low‐dose HRTEM and bright‐field (BF) scanning TEM (STEM) scanning moiré fringes (SMFs). Before acquiring HRTEM images of furosemide, a model system of crocidolite (asbestos) was used to determine the electron flux/fluence limits of low‐dose HR imaging for our scintillator‐based, complementary metal‐oxide semiconductor (CMOS) electron camera by testing a variety of electron flux and total electron fluence regimes. An electron flux of 10 e^−^/(Å^2^ s) and total fluence of 10 e^−^/Å^2^ was shown to provide sufficient contrast and signal‐to‐noise ratio to resolve 0.30 nm lattice spacings in crocidolite at 300 kV. These parameters were then used to image furosemide which has a critical electron fluence for damage of ≥10 e^−^/Å^2^ at 300 kV. The resulting HRTEM image of a furosemide crystal shows only a small portion of the total crystal exhibiting lattice fringes, likely due to irradiation damage during acquisition close to the compound's critical fluence. BF‐STEM SMF images of furosemide were acquired at a lower electron fluence (1.8 e^−^/Å^2^), while still indirectly resolving HR details of the (001) lattice. Several different SMFs were observed with minor variations in the size and angle, suggesting strain due to defects within the crystal. Overall BF‐STEM SMFs appear to be more useful than BF‐STEM or HRTEM (with a CMOS camera) for imaging the crystal lattice of very beam‐sensitive materials since a lower electron fluence is required to reveal the lattice. BF‐STEM SMFs may thus prove useful in improving the understanding of crystallization pathways in organic compounds, degradation in pharmaceutical formulations and the effect of defects on the dissolution rate of different crystal faces. Further work is, however, required to quantitatively determine properties such as the defect density or the amount of relative strain from a BF‐STEM SMF image.

## Introduction

For the pharmaceutical industry, obtaining atomic lattice resolution images of organic crystalline compounds can provide important information on the effects of formulation processing on drug properties. For example, milling is routinely used to decrease particle size and improve dissolution rates of poorly water‐soluble, active pharmaceutical ingredients (APIs) (Naik & Chaudhuri, 2015). As a result of this process, defects can be introduced into crystalline APIs. These defects are known to be sites that can initiate polymorphic phase transformations and hydrate formation (Byard *et al*., 2005; Koivisto *et al*., 2006; Eddleston *et al*., 2010; Eddleston & Jones, 2016). Additionally, crystalline defects at particular crystal faces can influence dissolution rates, due to a change in interfacial dissolution kinetics, governed by the energetics of the free surface (Macpherson & Unwin, 1995). In particular, dislocations are thought to be important drivers of etch pit formation during dissolution, leading to an increase in specific surface area and the formation of microdomains of higher surface energy (Perry *et al*., 2015; Adobes‐Vidal *et al*., 2016).

Bulk techniques such as powder X‐ray diffraction (pXRD), differential scanning calorimetry (DSC) and Fourier transform infrared (FTIR) spectroscopy are routinely used for solid‐state characterization of APIs; however, they are unable to assess nonperiodic structures on the nanoscale (Eddleston *et al*., 2010). Conventional transmission electron microscopy (CTEM) and scanning transmission electron microscopy (STEM) are two techniques that can provide this information, are complementary to the bulk techniques and also provide further information into the nanostructure of APIs (Ricarte *et al*., 2015, 2016).

The main challenge in using CTEM and STEM for analysis of pharmaceuticals is the damage caused to a specimen from the energy deposited by the electron irradiation, predominately via radiolytic processes (Egerton *et al*., 2004). Damage mitigation requires the use of low electron dose techniques to preserve the initial structure as much as possible. Damage proceeds under irradiation by a loss in structural order that typically follows an exponential form (Reimer & Spruth, 1982). At a given accelerating voltage, the characteristic or critical electron fluence (C_*F*_) over which a significant loss in order (1/e) will occur can be used as a measure of the electron beam sensitivity of a material (Henderson & Glaeser, 1985). A recent study measured the C_*F*_ of 20 chemically diverse but poorly water‐soluble APIs under 200 keV electron irradiation and found that the majority of compounds had a C_*F*_ less than 5 e^−^/Å^2^ (S'ari *et al*., 2018). In the past, HRTEM images of electron‐beam‐sensitive materials have been recorded on photographic film, and more recently direct electron detectors have been used to obtain HRTEM images at low dose (Murata *et al*., 1976; Smith & Fryer, 1981; Zemlin *et al*., 1985; Revol & Manley, 1986; Zhang *et al*., 2018). One example is by Zhang *et al*. (2018) where atomic resolution images of metal‐organic frameworks (MOFs) were obtained by HRTEM using a total electron fluence of 5 e^−^/Å^2^ (Zhang *et al*., 2018). The use of direct electron detectors increases the signal‐to‐noise ratio (SNR) and contrast in an image due to the high detector quantum efficiency (DQE) compared to conventional scintillator‐based detectors and this is key to enabling HRTEM at very low electron fluence.

Another technique that has been used to obtain HR, atomic lattice information at low electron fluence, without the need for a direct electron detector, is scanning moiré fringes (SMFs) in STEM (Su & Zhu, 2010; Naden *et al*., 2018; S'ari *et al*., 2019). SMFs arise from the interference between atomic plane spacings in a crystal lattice and spacings in a similarly sized reference lattice, produced by the scanning of the electron beam. The resulting generated pattern produces a magnified image of the crystal lattice, including any imperfections, and allows lower magnification acquisitions and therefore larger areas to be imaged at a lower electron fluence than would normally be required. Generally, SMFs have been explored using high‐angle annular dark‐field (HAADF) STEM imaging to obtain large‐area strain measurements in semiconductors and functional oxides (Su & Zhu, 2010; Kim *et al*., 2013a, b; Murakami *et al*., 2015; Ishizuka *et al*., 2017; Naden *et al*., 2018).

Here, we report on the use of HRTEM and SMFs captured using bright‐field (BF) STEM to acquire atomic lattice images of an electron‐beam‐sensitive pharmaceutical crystal, furosemide. Before acquiring HRTEM images of furosemide, a model system of crocidolite (asbestos) is used to determine the electron flux/fluence limits of low‐dose HR imaging for our scintillator‐based, CMOS electron camera by testing a variety of electron flux and total electron fluence regimes. The regime that provides sufficient SNR and contrast to resolve the larger atomic plane spacings and that falls within the C_*F*_ of furosemide is then used to collect HRTEM images. Finally, BF‐STEM and SMF images of defects within the furosemide lattice are obtained at fluences below C_*F*_ and the HRTEM and SMF results are qualitatively compared.

## Materials and methods

### Materials

Crocidolite, the fibrous form of the mineral riebeckite (COD ID: 9004132) and recognized as one of the six types of asbestos (also known as blue asbestos), was purchased from Agar Scientific Ltd and dispersed on a thin film for TEM (Hawthorne, 1978). This was used as a model system to test the minimum electron flux/fluence required to obtain HRTEM images on our CMOS camera, due to being relatively electron beam stable compared to organic crystals, and because it contains large lattice spacings in the range of 7–9.5 Å, similar to those found in furosemide. The organic crystal furosemide was examined during this study and has a C_*F*_ of 7 ± 4 e^−^/Å^2^ when measured at 200 kV accelerating voltage (S'ari *et al*., 2018). Both the inelastic and elastic scattering cross‐sections are affected by accelerating voltage and are proportional to $1/v^{2}$, where *v* is the incident electron speed (Egerton, 2014). Therefore, increasing the incident electron energy decreases the inelastic and elastic scattering cross‐sections. A decrease in the inelastic scattering cross‐section is preferable for samples that damage via radiolysis as this reduces the damage that occurs; the use of 300 kV rather than 200 kV has experimentally shown to increase $C\;\;{}_{F}$ by a factor of approximately 1.5 (Hayashida *et al*., 2006; Egerton, 2014; Cattle *et al*., 2015). However, the decrease in elastic scattering cross‐section reduces image contrast and limits the information obtainable per unit damage. This is particularly so for samples thinner than 60 nm, whereas for a thicker sample, i.e. >100 nm, there will be more scattering events and overall this will increase image contrast, up to a point (Egerton, 2014; Peet *et al*., 2019).

Furosemide (CCDC Refcode: FURSEM13) powder was provided by AstraZeneca (furosemide is a loop diuretic and antihypertensive drug, used to control cardiovascular and heart failure disease). Crystals of form I furosemide were prepared using the same method as Adobes‐Vidal *et al*. (2016) by dissolving furosemide powder in 0.5 mL of ethanol to make a 10 mM solution, which was then mixed with 3.5 mL of deionized water, this formed small crystals which were left to grow in the solution for 10 min (Babu *et al*., 2010; Adobes‐Vidal *et al*., 2016). Previous work by Adobes‐Vidal *et al*. (2016) confirmed by pXRD that the resulting crystals were furosemide form I. The crystals were then floated onto a carbon‐coated copper TEM grid and allowed to air dry before imaging. The unit cell parameters of both riebeckite and furosemide are shown in Table 1.

**Table 1:** Unit cell parameters for riebeckite and furosemide (Hawthorne, 1978; Babu *et al*., 2010)

	Riebeckite	Furosemide
**Crystal system**	Monoclinic	Triclinic
**Space group**	C 1/m 1	P‐1
**a/Å**	9.811	9.515
**b/Å**	18.013	10.448
**c/Å**	5.326	15.583
$\alpha {/}^{\circ }$	90	92.84
$\beta {/}^{\circ }$	103.68	107.09
$\gamma {/}^{\circ }$	90	116.75

### Equipment

All samples were examined in an FEI Titan^3^ Themis G2 operated at an accelerating voltage of 300 kV, equipped with a field emission gun (X‐FEG) operating at an extraction voltage of 4.5 kV and a monochromator. HR images and diffraction patterns were acquired using a Gatan OneView CMOS camera and an FEI BF‐STEM detector was used to acquire SMF images. The pixel size at each magnification and diffraction pattern had previously been calibrated using a standard of gold nanoparticles on graphite.

The electron flux in CTEM was controlled by adjusting the monochromator focusing lens and the C2 condenser lens and was set to 0.08 e^−^/(Å^2^ s) when searching for areas of interest at low magnification and acquiring selected area electron diffraction (SAED) patterns. In STEM, the total electron fluence per image was controlled by altering the magnification, reducing the probe current to 5 pA using the monochromator focusing lens and setting the dwell time per pixel to 10 µs. The electron flux and measured probe current in CTEM and STEM were based on a flu‐cam current reading which had been previously calibrated using a Faraday cup, and therefore reported uncertainties in the electron flux and probe current measurements are readout error from the flu‐cam. In TEM, this corresponds to ±0.01 e^−^/(Å^2^ s) and in STEM ±1 pA.

### Low‐dose HRTEM method

To achieve HRTEM images at low dose, it is important that the total electron fluence received by the specimen before capturing the image was controlled to stay well below C_*F*_ (thereby maximizing the available fluence or dose budget for the image acquisition). Here, an electron flux of 0.08 e^−^/(Å^2^ s) and BF‐TEM with a 30 mrad objective aperture were used to identify areas of interest. Once an area had been identified, the electron beam was positioned around the area of interest and blanked to reduce further irradiation. The magnification was increased to 115 kX and the C2 lens current was changed to a preset value that provided the desired electron flux. The electron beam was then unblanked and the objective lens focus quickly adjusted before acquiring an image using the electron counting mode of the CMOS camera, set to a desired total electron fluence. If the crystal was large enough, the objective lens focus was adjusted on a sacrificial area before image acquisition to reduce the amount of time needed to reach appropriate focus to reveal lattice fringes (approximately Scherzer defocus).

### Scanning moiré fringe method

As outlined earlier, SMFs are formed due to the interference between the scanning lattice produced by rastering the electron beam in STEM, which acts as the reference lattice (spacing *d*_*s*_), and the lattice fringes of a crystal (spacing *d*_*l*_). The size and orientation of the generated SMFs depend on *d*_*s*_, *d*_*l*_ and the angle between both lattices (θ) (Li *et al*., 2010; Su & Zhu, 2010; S'ari *et al*., 2019). The magnitude of d_*s*_ is equivalent to the pixel size of the image and can only take discrete values that depend on the magnification in STEM. When θ is equal to zero the resulting SMFs spacings are as large as possible and the angle between the SMFs and the scanning lattice, known as ϕ, is also equal to zero.

To align the scanning lattice and crystal lattice as parallel as possible (i.e. θ = 0), the scan direction in STEM was rotated. To calculate the angle required to rotate the scan direction, an SAED pattern of the crystal was recorded in CTEM mode, on the CMOS camera, using an electron flux of 0.08 e^−^/(Å^2^ s). The angle required to align the desired crystal lattice so that the lattice is aligned either horizontally or vertically in the STEM images was then measured. In addition to this angle, there is a difference in orientation between the CMOS camera used to acquire the diffraction pattern in TEM and the scan coordinates in STEM. This must also be taken into account when calculating the amount required to rotate the scan direction (S'ari *et al*., 2019).

Once an area of suitable (furosemide) crystal alignment had been identified and an SAED pattern acquired, the microscope was operated in BF‐STEM mode with a probe current of 5 pA (and the collection semiangle of the BF detector set to approximately half of the probe convergence semiangle of 11 mrad). The sample was brought close to focus at a magnification of 10 kX and the desired area was aligned in the centre of the field of view. The electron beam was then blanked and the magnification increased to that slightly lower than required to obtain SMFs. Either a sacrificial area of the sample, or a region on the carbon support film immediately adjacent to crystal, was then used to adjust focus using the Ronchigram of the probe parked just next to the intended image area, so as to limit the total electron fluence impinging on the desired area. The magnification was then increased and a BF‐STEM image containing SMFs was acquired.

## Results and discussion

### HRTEM of crocidolite

**Figure 1:**
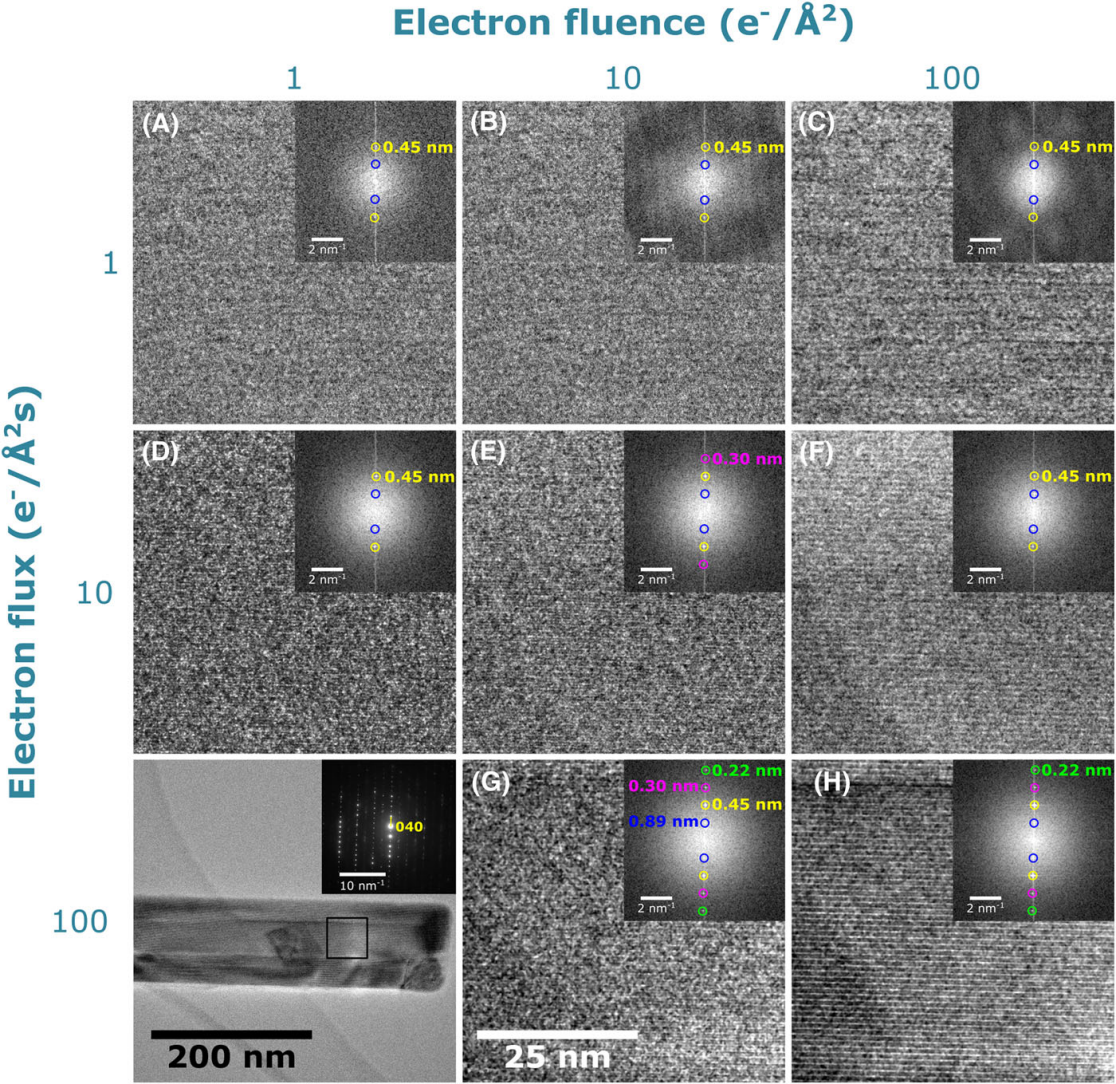
HRTEM of a crocidolite needle (low‐magnification image showing magnified area and SAED pattern inset in the bottom left of the panel), used as a model system to determine which electron flux and electron fluence regime would be best suited for clearly resolving lattice information at low dose. Images (A)–(C) were captured using an electron flux of 1 e^−^/(Å^2^ s) and show the same area of the crocidolite crystal but are exposed for a total electron fluence of 1, 10 and 100 e^−^/Å^2^, respectively. Images (D)–(F) were captured using an electron flux of 10 e^−^/(Å^2^ s) at a total electron fluence of 1, 10 and 100 e^−^/Å^2^, respectively. Finally, (G) and (H) were captured using an electron flux of 100 e^−^/(Å^2^ s) at a total fluence of 10 and 100 e^−^/Å^2^, respectively. Inset in images (A)–(H) is the corresponding FFT with visible lattice points highlighted.

HRTEM images acquired at total electron fluences of 1, 10 and 100 e^−^/Å^2^ and at three different electron fluxes 1, 10 and 100 e^−^/(Å^2^ s) of the same area of crocidolite are shown in Figure 1, alongside the fast Fourier transform (FFT) for each image. No image was acquired for an electron flux of 100 e^−^/(Å^2^ s) at a total fluence of only 1 e^−^/(Å^2^ s) due to the CMOS detector being unable to capture an image for such a short time. Instead, lower magnification image of the crocidolite needle highlighting the magnified area and the SAED pattern showing the (040) direction are shown in Figure 1 (bottom left).

Figures 1(G) and (H) were acquired at an electron flux of 100 e^−^/(Å^2^ s) at a total fluence of 10 and 100 e^−^/Å^2^, respectively. From the FFTs, the crocidolite (020), (040), (060) and (080) lattice spacings can be identified, equal to 0.89 ± 0.03, 0.45 ± 0.01, 0.30 ± 0.00 and 0.22 ± 0.00 nm, respectively. The (080) spot at 0.22 ± 0.00 nm is close to the Nyquist limit of 0.196 nm for a TEM image magnification of 89 kX, which corresponds to a calibrated pixel size of 0.098 nm, the total number of pixels being 4096 × 4096 and a field of view of 399.76 × 399.76 nm.

Figures 1(D)–(F) were acquired at an electron flux of 10 e^−^/(Å^2^ s) at a total fluence of 1, 10 and 100 e^−^/Å^2^, respectively. In all of these images, it is possible to resolve the (020) and (040) spacings; however, the (060) spacing could only be seen in the image in Figure 1(E). None of the images taken using a flux a 10 e^−^/(Å^2^ s) were able to sufficiently resolve the (080) spacing. Figures 1(A)–(C) were taken using an electron flux of 1 e^−^/(Å^2^ s) with a total fluence of 1, 10 and 100 e^−^/Å^2^, respectively. At this electron flux, only the larger (020) and (040) spacings could be resolved, although these can be difficult to see. Additionally, in Figures 1(B) and (C), artefacts appear in the background of the FFT, possibly due to the low signal and longer image integration time.

To quantitatively compare the quality of each image, the SNR and experimentally measured contrast, *F*(*g*)/*F*(0), in the FFT power spectrum was calculated and compared to the theoretical contrast by using the method described in Henderson *et al*. (1986) and Sader *et al*. (2010). Here, the SNR was calculated by measuring the maximum intensity at the expected peak position as well as the average background intensity from an adjacent area in the power spectrum. This background was then subtracted from the maximum and divided by the background to give the SNR, and these values are shown in Table 2. To be certain that a feature in the image is real, the SNR must be greater than 3–5 to satisfy the Rose criterion (Rose, 1974). The theoretical contrast was measured from the electron diffraction pattern and is given by $F\left(g\right)/F\left(0\right)=2\sqrt{I(g)/I(0)}$, where *I*(0) is the integrated intensity of just the zero‐order diffraction spot and *I*(*g*) is the integrated intensity of the first‐order diffraction spot minus the integrated intensity in an adjacent background area. The power spectrum was used to measure the experimental contrast, here *I*(0) was equal to just the intensity at the origin and I(g) was measured from the integrated intensity around the expected peak position minus the integrated intensity in an adjacent background area. Table 3 shows the percentage ratio between experimental image and theoretical diffraction values of F(g)/F(0).

**Table 2:** Signal‐to‐noise ratio of each *d*‐spacing in the images of crocidolite shown in Figure 1

		Signal‐to‐noise ratio							
hkl	d‐spacing (nm)	a	b	c	d	e	f	g	h
(080)	0.22 ± 0.00	1	2	1	22	4	3	211	174
(060)	0.30 ± 0.00	1	1	1	6	11	3	66	45
(040)	0.45 ± 0.01	3	5	3	100	110	21	320	642
(020)	0.89 ± 0.03	5	14	55	46	76	341	223	564

**Table 3:** Percentage ratio of experimental image and theoretical diffraction F(g)/F(0) values from each *d*‐spacing in the images of crocidolite shown in Figure 1

		Ratio of experimental (image) and theoretical							
		(diffraction) F(g)/F(0) values (%)							
hkl	d‐spacing (nm)	a	b	c	d	e	f	g	h
(080)	0.22 ± 0.00	3.3	1.7	0.5	2.9	0.9	0.2	2.7	1.3
(060)	0.30 ± 0.00	6.6	1.9	0.6	3.0	1.4	0.3	2.9	1.2
(040)	0.45 ± 0.01	12.3	4.7	1.9	23.3	10.3	1.3	12.5	10.7
(020)	0.89 ± 0.03	11.0	6.9	6.8	17.6	8.8	5.8	12.9	11.8

To explain the differences observed in the SNR at the various electron fluences and fluxes, it is useful to consider Eq. (1), which shows the parameters that affect the SNR of a detector:

(1) $$SNR=\frac{Jt\mathrm{DQE}}{\sqrt{Jt\mathrm{DQE}}+Dt+N_{r}^{2}}.$$

Here, *J* is the electron flux, *t* is the image integration time, DQE is the detector quantum efficiency, *D* is the dark current value and *N*_*r*_ is the read‐out noise. Between Figures 1(A)–(C), the electron flux is the same but the total electron fluence increases, and from Eq. (1), it would be expected that SNR would increase. This is the case for the SNR of the (020) spacing, whereas all other spacings are either below or on the edge of satisfying the Rose criterion. Similarly, there was a general increase in SNR between Figures 1(D) and (E), apart from the (080) spacing in image (D) where the SNR value was unexpectedly high at 22 compared to 4 in Figure 1(E). This higher SNR was measured from a single pixel and had a considerably higher intensity than the background at the expected peak position. The high intensity could be due to random fluctuations in the noise.

The SNR for the (040) and (060) spacings in Figures 1(E) and (F) decreases by a factor of 3–5 with an increase in total fluence, whereas the (020) spacing increases by a factor of 4. Likewise, in Figures 1(G) and (H), the SNR of the higher order spacings, (080) and (060), is higher at low total fluence, whereas the lower order spacings, (040) and (020), are higher at an increased fluence. This change in intensity may be caused by the increase in image integration time required to reach higher electron fluences, resulting in small change in sample orientation that can cause quite strong dynamical diffraction effects that may affect the reflection intensities during the tilt. The ratio between experimental image and theoretical diffraction F(g)/F(0) values also generally decreases with increasing total fluence (for a constant electron flux) and is consistent for all images. Similar results for values of image contrast are shown by Henderson and Glaeser (1985), where the reported F(g)/F(0) of beam‐sensitive specimens drastically decreased due to specimen movement (Henderson & Glaeser, 1985).

Based on the results presented for crocidolite, the best quality lattice images obtainable at low dose should use as high an electron flux as possible, to reduce the time in which the specimen can move and be acquired at a total electron fluence close to the critical fluence to provide as much signal as possible. From this and the previously measured C_*F*_ of furosemide (7 ± 4 e^−^/Å^2^ at 200 kV and approximately 10 ± 6 e^−^/Å^2^ at 300 kV), an electron flux of 10 e^−^/(Å^2^ s) and total fluence of 10 e^−^/Å^2^ was selected. These values should minimize the overall exposure time when acquiring an image and still allow for a small amount of fluence (and therefore time) to find and focus the crystal. In theory, using a higher flux and therefore shorter integration time to reach the desired total fluence would provide better SNR and contrast; however, it would leave insufficient time to focus ahead of image acquisition.

### HRTEM of furosemide

**Figure 2:**
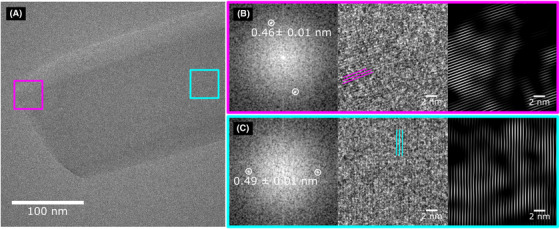
(A) HRTEM of furosemide, collected using an electron flux of 10 e^−^/(Å^2^ s) and an electron fluence of 10 e^−^/Å^2^ at a magnification of 115 kX. (B) The (red) cropped area from (a) showing: the FFT with d‐spacings of 0.46 ± 0.01 nm, the raw image and the Fourier‐filtered image; (C) (blue) cropped area showing: the FFT with d‐spacings of 0.49 ± 0.01 nm, the raw image and the Fourier‐filtered image.

Figure 2 shows an HRTEM image of furosemide taken using an electron flux and fluence of 10 e^−^/(Å^2^ s) and 10 e^−^/Å^2^, respectively. From the overall image (in Fig. 2A), two different areas are further magnified on the crystal to more easily show the lattice fringes. Figure 2(B) shows the FFT of the magnified area showing spots that are equal to 0.46 ± 0.01 nm. The raw magnified image highlighting where the fringes appear and a Fourier‐filtered image formed using the 0.46 ± 0.01 nm spots in the FFT and then an inverse FFT is also shown. Similarly, Figure 2(C) shows the FFT with spots measured at 0.49 ± 0.01 nm, the raw magnified image and the Fourier‐filtered image. Table 4 shows the possible hkl values that both the 0.46 ± 0.01 and 0.49 ± 0.01 nm spacings could be assigned to based on the measured size and angle between the spots which was $70.6\pm 1.0^{\circ }$.

**Table 4:** Potential pairs for the hkl values of the 0.46±0.01 nm and 0.49±0.01 nm spots measured from the FFTs in Figure 2 of furosemide form I

hkl	d‐Spacing (nm)	hkl	d‐Spacing (nm)	Angle (°)
		
0.46 ± 0.01		0.49 ± 0.01		70.6 ± 1.0
$\left(0\bar{2}1\right)$	0.46	$\left(\bar{1}03\right)$	0.50	69.8
(102)	0.46	$\left(\bar{1}03\right)$	0.50	69.0
(102)	0.46	$\left(1\bar{2}1\right)$	0.49	70.7
$\left(\bar{1}13\right)$	0.46	$\left(11\bar{2}\right)$	0.49	70.1
$\left(\bar{2}12\right)$	0.45	(003)	0.49	71.2

Other small regions of the crystal also appear to be crystalline from the FFT although, when these areas are cropped and magnified, no visible lattice fringes appear in the raw image. This is due to the unit cell of the crystal being fairly large and the fact that the FFT is averaging over many unit cells, increasing the SNR and allowing spots to be visible in the FFT, even though the lattice is not directly apparent in the image (Sader *et al*., 2010).

Applying a Fourier filter around the spots in the FTT from Figures 2(B) and (C) and then carrying out an inverse FFT remove most of the noise in the image which can then be used to more easily identify fringes and possible defects. From the Fourier‐filtered images, there do not appear to be any defects within the lattice, although the fringes appear in patches and are not found uniformly across the entire particle. This is most likely due to the effects of electron beam damage, since the $C\;\;{}_{F}$ for furosemide is close to the total fluence used to acquire the image. In addition, the crystal had already been exposed to the electron beam while searching for potential areas and focusing; prior to capturing the image. Therefore, consuming part of the limited electron dose budget available. All of these factors increase the difficulty in obtaining and interpreting the results of HRTEM images of furosemide at low dose. To obtain more representative results, a large number of areas need to be sampled to find suitable regions that are thin, in focus and have not been overly exposed and therefore damaged.

### Scanning moiré fringes of furosemide

**Figure 3:**
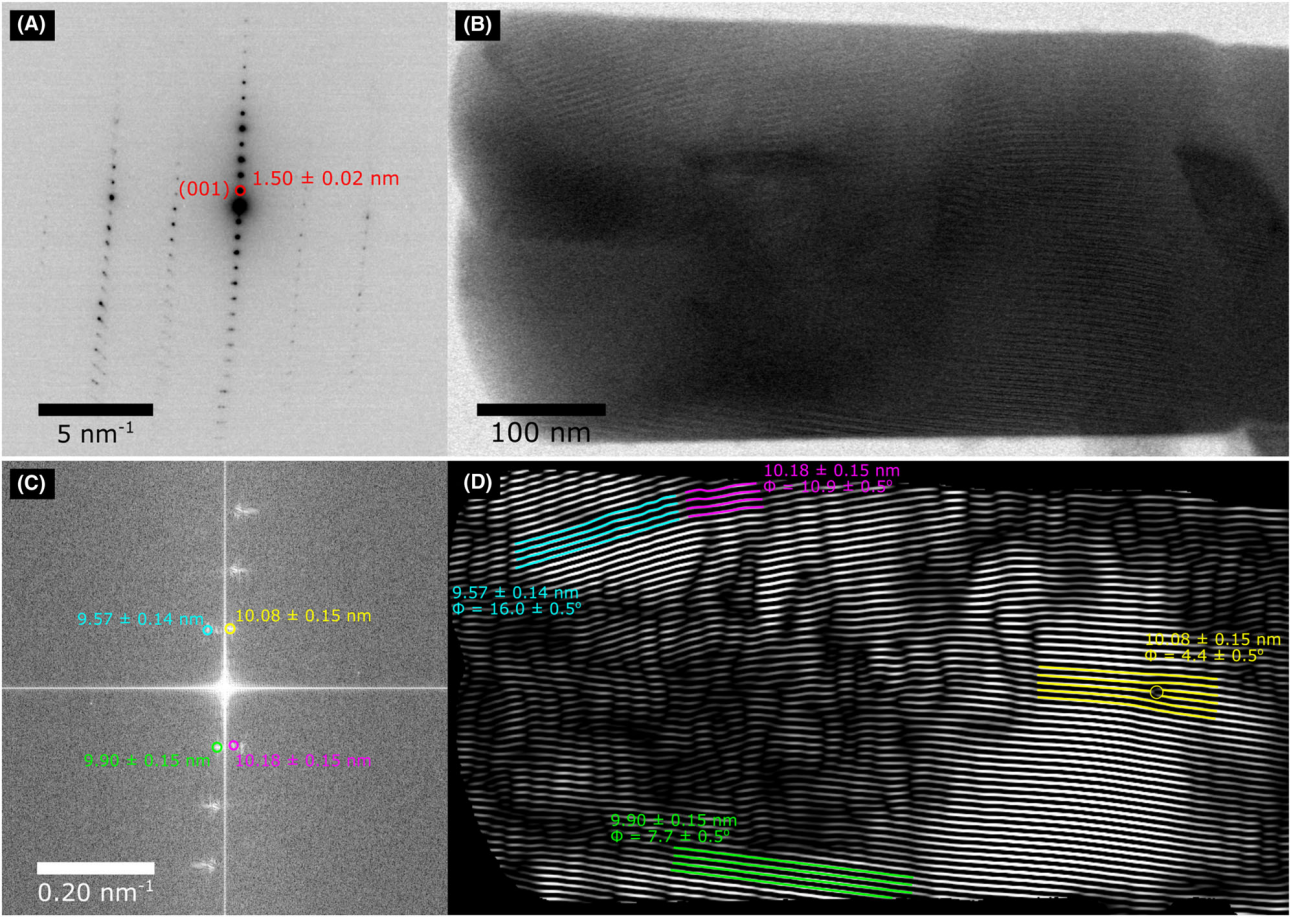
(A) SAED pattern of the furosemide crystal, the highlighted spacings, was measured at 1.50 ± 0.02 nm similar to the (001) reflection of furosemide form I. (B) SMF image which was a result of the interference between the (001) d‐spacing and the 1.32 nm scanning lattice in STEM. This was acquired at a magnification of 74 kX using a total electron fluence of 1.8 e^−^/Å^2^. (C) FFT of the SMF image and (D) inverse FFT of (C) after applying Fourier filter around the first‐order spacings. Variations in ϕ and the size of the SMF are labelled.

Figure 3(A) shows the SAED pattern of a furosemide crystal that was subsequently imaged with a systematic row, and the first‐order spacings of this row were measured at 1.50 ± 0.02, which is close to the (001) reflection equal to 1.46 nm. The SMF image taken from this crystal is shown in Figure 3(B) at a magnification of 74 kX and was a result of arranging the (001) lattice spacing (of 1.50 ± 0.02 nm) to align and interfere with the STEM scanning lattice (of 1.32 nm). The total electron fluence used to acquire the image was 1.8 e^−^/Å^2^. Figure 3(C) shows the FFT of the SMF image. Several different first‐order moiré spots are highlighted including those corresponding to fringe spacings of 9.57 ± 0.14, 10.08 ± 0.15, 9.90 ± 0.15 and 10.18 ± 0.15 nm. The position of these fringes can be identified in real space by applying a Fourier filter and carrying out an inverse FFT, as shown in Figure 3(D). The range of similar sized SMFs and changes to ϕ (the angle between SMFs and scanning lattice) indicate that θ is nonzero and small variations occur in the size of the (001) spacing or orientation, suggesting the presence of strain in the crystal. In addition, defects that appear similar to edge dislocations can be identified within the lattice, such as the one located in the fringes highlighted at 10.07 ± 0.15 nm and ϕ = $4.4\pm 0.5^{\circ }$. These are only interpretable as edge dislocations in the crystal lattice if θ = 0, such that the generated SMFs are translational.

**Figure 4:**
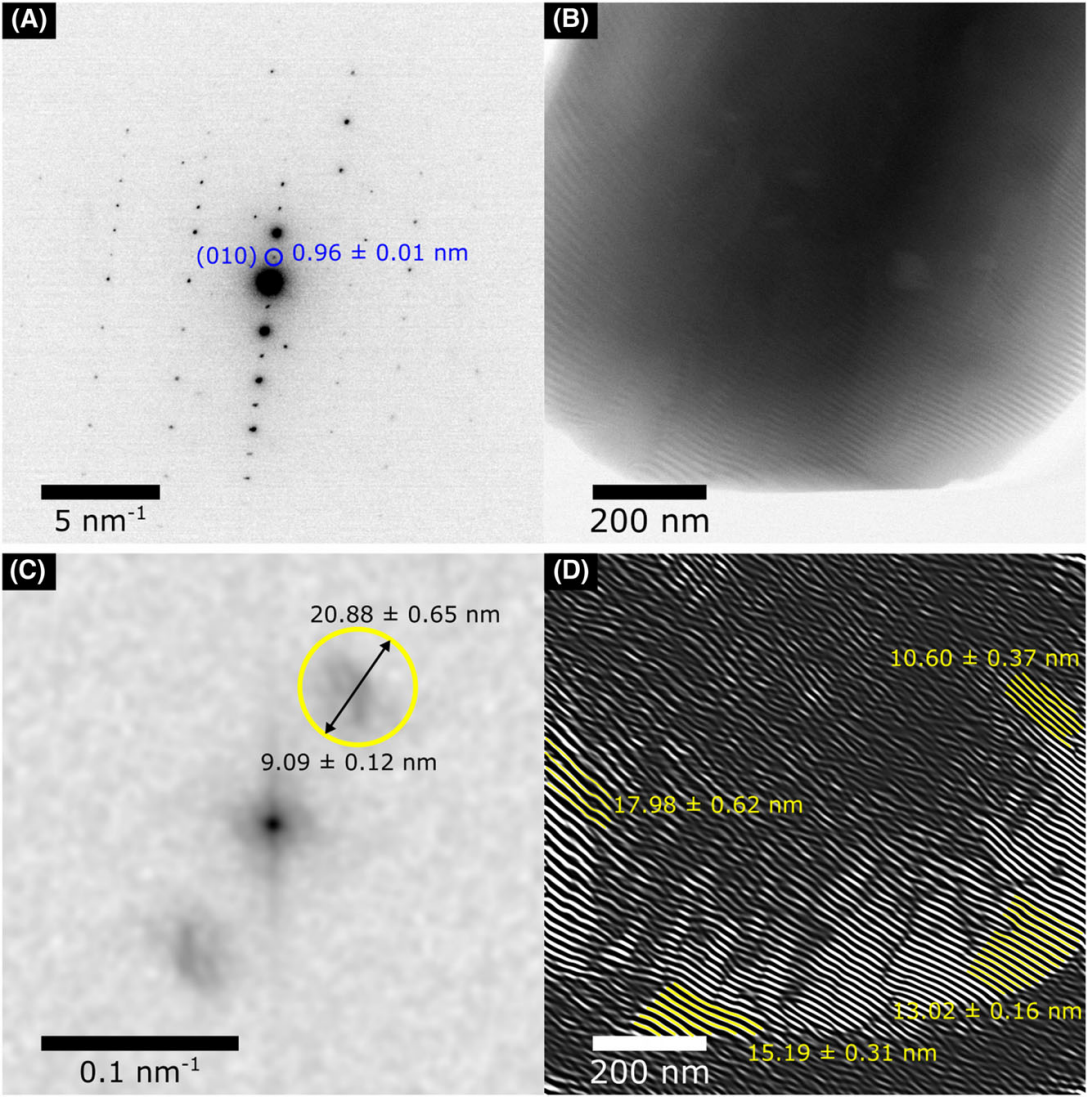
(A) SAED pattern of the furosemide crystal, the highlighted spacings, was measured at 0.96 ± 0.01 nm similar to the (010) reflection of furosemide form I. (B) SMF image which was a result of the interference between the (010) d‐spacing and the 0.93 nm scanning lattice in STEM. This was acquired at a magnification of 105 kX using a total electron fluence of 3.6 e^−^/Å^2^. (C) FFT of the SMF image, the highlighted area, shows the size of the Fourier filter applied. (D) Inverse FFT of (C) after applying Fourier filter around the highlighted region.

Figure 4 shows SMFs from another furosemide crystal; however, instead of producing SMFs from the (001) reflection and 1.32 nm scanning lattice, the (010) reflection equal to 0.96 ± 0.01 nm is used, as measured from the SAED in Figure 4(A), and 0.93 nm scanning lattice. The SMF image in Figure 4(B) was collected using a total electron fluence of 3.6 e^−^/Å^2^. A diffuse area of higher than background intensity can be seen in Figure 3(C), and this area contains information on spacings between 9.09 ± 0.12 nm and 20.88 ± 0.65. The inverse FFT of the highlighted region is shown in Figure 4(D), where several fringes of different sizes can be seen and some are highlighted, these being 17.98 ± 0.62, 15.19 ± 0.31, 13.02 ± 0.16 and 10.60 ± 0.37 nm. The large variations in SMF sizes suggest that the original lattice contains a larger number of defects or is more highly strained when compared to the smaller variations seen in the SMF sizes for the (001)‐oriented crystal (Fig. 3). Similarly, previous work showing SMFs of the (001) lattice spacing of furosemide form I shows little variation in the size of the SMFs (S'ari *et al*., 2019).

Although the Nyquist limit decreases when using SMFs relative to directly imaging the crystal lattice with high‐magnification BF‐STEM imaging, the electron fluence used is much lower and the fringes are magnified. For example, here the first‐order SMFs for the (001) reflection are approximately 10 nm which is within 3.8 times the Nyquist limit of 2.64 nm at 74 kX magnification using a pixel size of 1.32 nm, with a total number of pixels 1024 × 1024 and a field of view of 1351 × 1351 nm.

It would not be possible to directly image the (001) lattice at this magnification by BF‐STEM since the pixel size is similar (1.32 nm). To achieve a direct (001) lattice image by BF‐STEM, a magnification with a scanning pixel size of 0.33 or 0.23 nm would be required to sufficiently resolve the fringes, corresponding to an electron fluence of 28.7 or 59.1 e^−^/Å^2^, respectively. This assumes that the probe current and pixel dwell time are kept the same, i.e. at least 2–3 larger than the C_*F*_ of furosemide.

When comparing the SMF image of the (001) reflection (Fig. 3) to the HRTEM image (Fig. 2), the former provides a larger field of view (1352 nm^2^ compared to 311 nm^2^) and uses less than 1/5 of the electron fluence required by HRTEM. As mentioned previously, the Nyquist limit decreases for the SMF images (2.64 nm), compared to the HRTEM images due to the camera having a higher sampling rate and smaller pixel size at the magnification used (0.152 nm at 115 kX and 0.196 nm at 89 kX). However, as shown in the crocidolite results (Fig. 1), the SNR closest to the Nyquist limit is generally less than 5 at an electron fluence of 10 e^−^/Å^2^. This is due to a combination of low fluence, the DQE of the detector and the modulation transfer function (MTF), which decreases the contrast and SNR at higher spatial frequencies (Sader *et al*., 2010). In the BF‐STEM scintillator‐photomultiplier detectors, the DQE is generally much higher compared to scintillator‐based charged‐coupled device (CCD) and CMOS cameras which increases the contrast and SNR, and also there is no MTF due to the image being collected sequentially point by point (Engel *et al*., 1981; Meyer & Kirkland, 2000). Larger areas of the crystal appear to be crystalline in the SMF image, suggesting that less damage has occurred consistent with the lower electron fluence required for acquisition. Therefore areas that are apparently defective or exhibit lattice strains by SMFs are more likely to be real, rather than artefacts originating from electron beam damage.

The main difficulty currently with the SMF images collected here is quantifying parameters such as the number of defects or the strain in the crystal and comparing the amount of strain in different *d*‐spacings. Other studies that use SMF on semiconductors and functional oxides have applied geometric phase analysis (GPA) as a method to assess strain and lattice rotation from the spatial variations in moiré fringe separations (Hÿtch *et al*., 1998; Kim *et al*., 2013b; Naden *et al*., 2018). This technique should prove useful for future analysis of the relationship between defect density or amount of strain present at a particular crystal face, for example, between the (001) and (010) faces and the known dissolution properties of that face for furosemide specifically, and pharmaceutical compounds in general (Adobes‐Vidal *et al*., 2016).

## Conclusion

In this study, HR lattice images of furosemide, an electron‐beam‐sensitive pharmaceutical crystal, have been acquired using two different methods: low‐dose HRTEM and BF‐STEM SMFs. For CTEM, an electron flux of 10 e^−^/(Å^2^ s) and total fluence of 10 e^−^/Å^2^ were shown to provide sufficient contrast and SNR to resolve 0.30 ± 0.00 nm lattice spacings in crocidolite (asbestos) at 300 kV and these parameters were used to image furosemide which has a C_*F*_ of ≥ 10 e^−^/Å^2^ at 300 kV. The HRTEM image suggests that the majority of the crystal is amorphous yet electron diffraction suggests this not to be the case and we believe that the acquisition fluence for imaging was sufficiently damaging so as to leave only a small proportion of the crystal still exhibiting lattice fringes. In addition, to successfully observe lattice fringes, the TEM image must be acquired from an area that is sufficiently thin and in focus, both of which can be difficult to determine at low electron flux. A small proportion of the total dose budget available before damage onset is also used to identify areas and capture an electron diffraction pattern to determine the orientation of the crystal, prior to HR imaging.

BF‐STEM SMF patterns are produced via interference between specific atomic plane spacings in a crystal and an STEM scanning lattice of similar size, causing interference and the lattice fringes and defects in the crystal to be magnified. This allows images to be acquired at a lower magnification, and therefore, lower electron fluence across a larger field of view, than direct imaging of the crystal lattice by BF‐STEM, whilst still indirectly resolving HR details. From the SMF image acquired of the (001) reflection of furosemide, at an electron fluence of 1.8 e^−^/Å^2^, several different SMFs can be seen with minor variations in the size and angle between the SMFs and the scanning lattice. These changes suggest that there is some strain within the crystal resulting in small differences in the size or orientation of the (001) spacing. Although the (010) reflection exhibited larger variations in the size of the SMFs, suggesting higher amounts of lattice strain. The cause of this strain may be due to defects within the crystal.

BF‐STEM SMFs appear to be more useful than BF‐STEM or HRTEM (with a CMOS camera) for imaging the crystal lattice of very beam‐sensitive materials since a lower electron fluence is required to reveal the lattice. BF‐STEM SMFs may thus prove useful in improving the understanding of crystallization pathways in organic compounds, degradation in pharmaceutical formulations and the effect of defects on the dissolution rate of different crystal faces. However, further work is required to quantitatively determine properties such as the defect density or amount of relative strain from a BF‐STEM SMF image. One method that has previously been applied to SMF image of other materials is GPA which has been used to assess the strain and lattice rotation from the spatial variations in moiré fringe separations.
